# Estrogen as a Neuroprotectant in Both Sexes: Stories From the Bird Brain

**DOI:** 10.3389/fneur.2020.00497

**Published:** 2020-06-23

**Authors:** Colin J. Saldanha

**Affiliations:** Departments of Neuroscience, Biology, Psychology & The Center for Behavioral Neuroscience, American University, Washington, DC, United States

**Keywords:** astrocyte, songbird, estradiol (17ß-estradiol), inflammation, neuroplasticity

## Abstract

Estrogens such as estradiol (E2) are potent effectors of neural structure and function via peripheral and central synthesis. In the zebra finch (*Taeniopygia guttata*), neural E2 synthesis is among the highest reported in homeotherms due to the abundant constitutive expression of aromatase (E-synthase) in discrete neuronal pools across the forebrain. Following penetrating or concussive trauma, E2 synthesis increases even further via the induced expression of aromatase in reactive astrocytes around the site of damage. Injury-associated astrocytic aromatization occurs in the brains of both sexes regardless of the site of injury and can remain elevated for weeks following trauma. Interestingly, penetrating injury induces astrocytic aromatase more rapidly in females compared to males, but this sex difference is not detectable 24 h posttrauma. Indeed, unilateral penetrating injury can increase E2 content 4-fold relative to the contralateral uninjured hemisphere, suggesting that glial aromatization may be a powerful source of neural E2 available to circuits. Glial aromatization is neuroprotective as inhibition of injury-induced aromatase increases neuroinflammation, gliosis, necrosis, apoptosis, and infarct size. These effects are ameliorated upon replacement with E2, suggesting that the songbird may have evolved a rapidly responsive neurosteroidogenic system to protect vulnerable brain circuits. The precise signals that induce aromatase expression in astrocytes include elements of the inflammatory cascade and underscore the sentinel role of the innate immune system as a crucial effector of trauma-associated E2 provision in the vertebrate brain. This review will describe the inductive signals of astroglial aromatase and the neuroprotective role for glial E2 synthesis in the adult songbird brains of both sexes.

The effects of estrogens such as 17β-estradiol (E2) on the structure and function of the vertebrate central nervous system (CNS) are well known (1–6). These include organizational effects such as the masculinization and feminization of neural circuits perinatally (1, 5, 7), organizational and activational effects during adolescence [reviewed in (8)], and activational effects on a diverse set of physiological endpoints during adulthood including, but not limited to, reproductive and aggressive behaviors, cognition, mood, motor control, and mood [see (9)]. We have more recently learned that the influence of this steroid extends even further than the physiology of the normal brain and potently modulates many processes involved in pathological conditions such as traumatic brain injury (TBI).

## Influence of E2 on the Injured Brain

Traumatic brain injury is defined in the clinical realm as a disruption in the normal function of the brain caused by percussive, concussive, or penetrating head injury. The incidence of TBI is strongly sexually dimorphic and male biased: a demographic characteristic attributed to higher rates of risky behavior in younger males [see (10) for review]. Following TBI, however, the predicted outcome and recovery of females are better than those of males (11). The underlying reason for this is hinted at by the observation that premenopausal women and those on hormone replacement have a lower risk of neurotraumatic events such as stroke, compared to the respective groups of age-matched men (12, 13). Following TBI in humans, both E2 and testosterone (T) decreased in the cerebrospinal fluid (CSF) over time. Importantly, a higher E2/T ratio was associated with lower mortality and better scores on the Glasgow Outcome Scale (GOS) 6 months after TBI (14). It is noteworthy that aromatase gene expression itself has been implicated in human TBI. More specifically, three single-nucleotide polymorphisms on the aromatase gene are associated with worse GOS-6 scores, suggesting that the expression of aromatase following TBI may be associated with differences in clinical outcomes post-TBI (14). The location of altered aromatase gene expression and the source of steroids in the CSF are unknown, but the pattern of data suggests the possibility that ovarian steroids may protect the brain from injury and/or damage and perhaps may even accelerate recovery.

Among the several steroids synthesized in the vertebrate ovary, E2 appears to be a powerful neuroprotectant as evidenced by multiple studies, using different types of TBI, in many vertebrate species. In rats, gerbils, and mice, females respond more favorably to medial carotid artery occlusion (MCAO) and other experimental inducers of ischemia (15–17). More recently, in mice subjected to controlled cortical impact, males demonstrated larger lesions compared to females (18). All these effects are apparently linked to circulating ovarian steroids because MCAO causes greater neural damage when it is conducted during metestrus compared to estrus, times of the rodent ovarian cycle when circulating E2 levels are low and high, respectively (15). In addition, infarct sizes increase following ovariectomy, and damage is exacerbated further the longer the animal is deprived of ovarian estrogens (19). The data demonstrate a neuroprotective effect of peripheral E2 across several species and types of damage. While it is true that all the aforementioned effects of E2 on the normal and injured brain reflect influences due to circulating levels of this steroid, there is excellent support for the notion that centrally synthesized E2 is a critical modifier of neurophysiological variables in both the normal and the injured CNS.

## Central Aromatization and the Normal Brain

The developing, juvenile, adult, and aging brains of mammals and birds are exquisitely sensitive to neural E2 synthesis [(1, 5, 7, 20–22); see (4) for review]. Much more recently, however, technological and conceptual developments have helped to reveal critical roles for central aromatization on other complex behaviors such as spatial memory in birds, rodents, and marmosets (23–26); seizure activity in rodents (27); and auditory perception and singing behavior in birds (28, 29).

The development of molecular, immunocytochemical, and ultrastructural tools to study the central expression of aromatase *in situ* revealed that with the exception of some teleost fish (30, 31) the expression of this enzyme is neuronal in the vast majority of species studies across all classes of vertebrates (32–41). Taken together, there is excellent reason to consider central, constitutive aromatization in neurons as key in the regulation of multiple physiological and behavioral endpoints in multiple vertebrate species.

## Glial Aromatization and the Injured Brain

The songbird and rodent brains, however, have an additional source of E2, one that is revealed following perturbation of the neuropil via multiple insults including excitotoxicity, concussive injury, penetrating injury, or edema. Specifically, aromatase expression can be induced in astrocytes at and around the site of brain damage in mice, rats, and zebra finches (*Taeniopygia guttata*) following all the types of injury mentioned [(42, 43); see (44)]. Importantly, this induction has been documented in the brains of both sexes [see (44–47) for review]. In rats and zebra finches, immunocytochemical studies using astrocytic markers and antibodies specific to aromatase reveal that injury-associated induction of aromatase appears localized to the area of damage and is limited to astrocytes and radial glia (42, 43, 48–50). It is important to point out, however, that, to the best of my knowledge, no study has specifically reported on changes in neuronal aromatase expression following neurotrauma in any species [but see (51)]. Because much of this special issue focuses on neurotrauma and neuroprotection in mammalian systems, to avoid redundancies, the rest of this review will focus on the induction, sex-specific expression, and consequences of glial aromatization in the zebra finch brain, but will mention similarities and differences between songbirds and rodents. We begin with a discussion about the induction of glial aromatase with emphasis on sex-specific mechanisms. We then describe the neuroprotective mechanisms of glial E2 provision highlighting some interesting sexually monomorphic and dimorphic patterns.

## The Songbird Model in the Neuroendocrinology of Brain Injury

The songbird has proven an invaluable animal model for studies of sexual differentiation (7), sex differences in brain and behavior (52), and the neural synthesis of estrogens (53). The obvious sensitivity of the songbird brain to locally synthesized E2 makes it, yet again, a perfect model toward understanding the role of centrally synthesized steroids on neuroplasticity. In our laboratory, we employ penetrating brain injury as a model toward understanding TBI in the songbird model. The vast majority of experiments in our laboratory are conducted, “within subject,” with contralateral telencephalic lobes of the finch brain treated as the experimental or control condition. In addition to halving the number of animals necessary for each study, this yields several additional advantages in experimental design, conduct, and interpretation. First, the injection needle used to deliver independent variables, such as inhibitors, antagonists, or cofactors itself, is the mechanical injury under study. It is therefore possible to study the effects of these variables both during and after the physical insult. Second, any observed differences between telencephalic lobes can be safely attributed to central effects and not those reflective of circulating factors. Third, because aromatase is a membrane-bound, nondiffusible protein [see (54, 55)], changes in the expression of this enzyme and differences between hemispheres, at least during the early stages postdamage, may be judged as independent and unlikely because of the influence of the contralateral lobe. Finally, because the product of aromatization is a lipophilic steroid, a conservative explanation of any lack of difference between lobes can be hypothesized to reflect diffusion and equilibration of E2 across the brain. This allows for the possibility that lessening the severity of injury, dose of experimental manipulation, and/or duration following the injury may reveal specific effects. As described below, this model has proven invaluable in testing specific hypotheses about the induction and influence of injury-induced aromatization.

## Inflammation Induces Aromatase Expression

There is a host of peripheral and central responses to TBI [see (56–58)]. Of these, perhaps one of the earliest, dramatic, and long-lasting is the activation of the innate immune system including the inflammatory response [see (59, 60)]. As such inflammatory processes themselves may play an inductive role in the expression of aromatase following brain damage. Consequently, our laboratory has focused on inflammatory signaling as one candidate that may be well-positioned as an inducer of astrocytic aromatase.

We reasoned that the induction of an inflammatory state with minimal mechanical damage to the neuropil would be helpful. Contralateral lobes of the zebra finch were exposed to either phytohemagglutinin (PHA) or saline. Importantly, the treatments were dripped onto the brain surface, thereby making mechanical penetration unnecessary (61). Astrocytic aromatase expression was abundant and confined to the lobe treated with PHA with no glial aromatase detectable on the saline-treated lobe. In contrast, the expression of neuronal aromatase was bilateral and similar across lobes suggesting a specific effect of PHA on glial aromatase expression (61). Finally, in an attempt to ascertain the specificity of the inductive signal responsible for the observed lateralized effect on glial aromatase, we measured the number of apoptotic cells in the lobe treated with PHA and compared it to one subjected to a penetrating injury. While abundant apoptosis was observed in the injured lobe, no apoptosis was detectable in the lobe exposed to PHA (61). This strongly suggested that the induction of aromatase could be induced by inflammatory signaling bypassing those associated with mechanical damage *per se*.

Of the many signals associated with the inflammatory cascade, we have focused our studies on the cytokines and the enzyme cyclooxygenase (COX). In our hands, injury causes a rapid increase in the cytokines TNF-α and IL-1β and the transcription of both COX1 and COX2 within hours (62, 63). We have capitalized on these changes in expression and directly measured the product of COX activity, the prostanoid prostaglandin E2 (PGE2). Indeed, the neural levels of PGE2 are dramatically increased following penetrating injury in the finch and have provided us with a powerful index of neuroinflammation and its stimulatory role in injury-induced aromatase expression. To test this hypothesis directly, we have conducted a systematic series of experiments that, in addition to revealing the mechanisms associated with the induction of glial aromatase and the neuroprotective effect of glial E2 provision, have suggested important sex-specific pathways that may prove crucial in the development of targeted therapies for TBI and neural damage in general.

## Sex Differences in the Induction and Action of Glial E2 Synthesis

**Figure 1:**
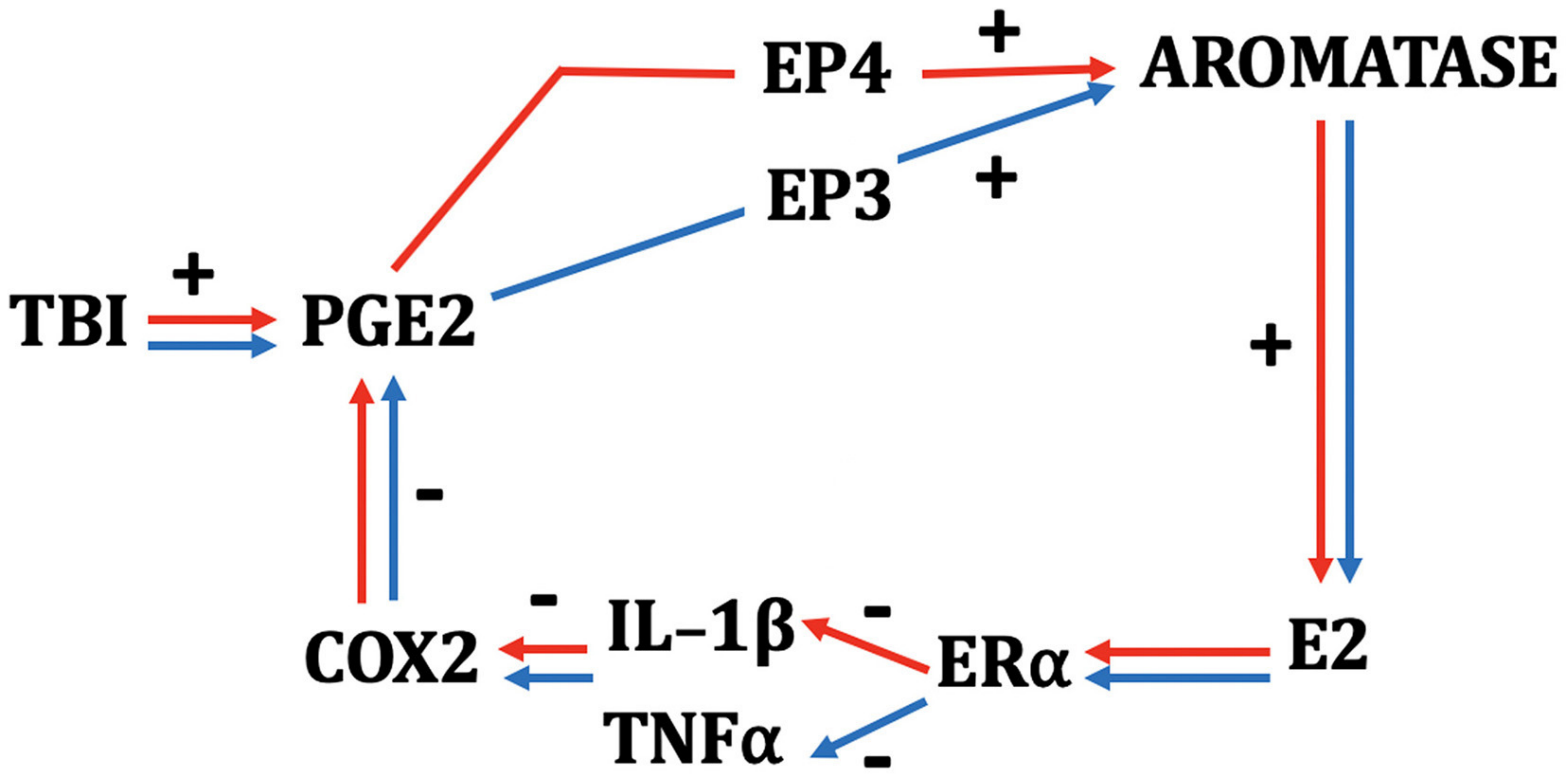
Schematic depicting the interactions among TBI, neuroinflammatory indices, and neurosteroidogenesis in female (red) and male (blue) zebra finches. While many components of the schematic are monovalent between the sexes, PGE2 results in a more rapid upregulation of aromatase in females and depends on signaling via the EP4 receptor. Males show a slower increase in aromatase via PGE2 action on the EP3 receptor (64, 65). E2 acts on ERα in both sexes and downregulates COX2 by actions predominantly on IL-1β in females and TNF-α in males.

Our early work on brain injury and aromatase expression in the songbird was restricted to male animals (43, 49, 51). The reason for this was because, in males of this species, the brain seems to be the major if not the only source of central and peripheral E2 (53). This approach proved to be shortsighted, as our first foray into understanding the female response to penetrating brain injury revealed that females upregulate glial aromatase more quickly than males (64). More specifically, while aromatase-positive glia are detectable around the injury site ~4 h after injury in the female, these cells are not reliably detected in the male until 12 or 18 h postdamage [see (64); Figure 1]. While the reason for this difference is yet unknown, it is important to state that no sex difference is detectable 24 or 48 h following a penetrating injury (49, 66, 67). The more rapid induction of aromatase following injury does not appear specific to a particular brain area, as a similar female-biased sex effect occurred following penetrating injury to the zebra finch cerebellum (68).

The more rapid response to injury in females has important implications when we began to study the mechanisms responsible for the induction of aromatase in astrocytes. As mentioned prior, there is good reason to hypothesize that elements of the inflammatory cascade, such as PGE2, may be excellent inducers of aromatase (see above). We therefore administered indomethacin, a nonspecific COX-1/2 inhibitor or vehicle into contralateral telencephalic lobes during a penetrating brain injury in adult male and female zebra finches (63). Subjects were killed either 6 or 24 h postinjury. To determine the efficacy of our manipulation, PGE2 levels were measured and at both time points, and indomethacin decreased the levels of this prostanoid in both sexes. In addition, COX-inhibition via indomethacin decreased aromatase expression and E2 content in both sexes, but this effect is detectable in temporally distinct patterns between the sexes. In females, the sex with the more rapid upregulation of injury-associated aromatase (see above), the influence of indomethacin is observed 6 h postinjury; however, at this time point, there is no sign of injury-induced aromatase expression in males. At 24 h postinjury, however, when the vehicle-treated lobes of males show dramatic increases in aromatase and E2, this is severely inhibited in the lobe treated with indomethacin (63, 65). The data strongly suggest that the inductive mechanism underlying astrocytic aromatase expression is similar between the sexes.

Sex differences are also revealed in the mechanism that may underlie the stimulatory role of PGE2 on aromatase expression. Pedersen et al. (65) have suggested that while EP3 receptors are necessary for the induction of aromatase and E2 following TBI in males, this effect is EP4 dependent in females (65). Taken together, these data strongly suggest that aspects of injury-induced inflammatory signaling are, in part, responsible for the induction of E2 following brain damage in both sexes, although the factors that sustain injury-induced aromatase expression in either sex are unknown. In both sexes, however, the product of aromatization is available at high levels to mediate the CNS response to trauma.

These data are in good agreement with studies conducted in mammalian systems both *in vitro* and *in vivo*. More specifically, inflammatory signals including IL-6 and PGE2 increase aromatase expression in breast cancer cells and benign cultures of breast cells *in vitro* (69–72). This remains true *in vivo*, at least in the normal developing brain. Aromatase expression and activity, as well as E2 levels, are all increased in the developing rat cerebellum following administration of PGE2 (73). In agreement, inhibition of the PGE2 synthetic enzyme, COX, causes a decrease in cerebellar aromatase and E2 levels (73, 74). These data underscore the viability of signals associated with inflammation as candidates that may regulate injury-induced aromatization in the brain.

The mechanisms associated with the more rapid induction of aromatase in females are unknown. It is possible that penetrating injury causes a more rapid induction of inflammation in females, resulting in a more rapid induction of aromatase. Alternatively, COX activity in females could be more responsive to cytokine signaling, and/or the aromatase gene in female astrocytes may be more responsive to PGE2 relative to males. Investigating these possibilities requires a very fine analysis of the time course of multiple inflammatory and steroidogenic profiles following injury in both sexes. These studies are ongoing in our laboratory.

The induction of aromatase expression specifically in glia around the injury site is also intriguing. We have long known that TBI is associated with rapid gliosis. However, the specific mechanisms that result in astrocytic aromatase expression (as opposed to all neural sources of aromatase) are unknown at the present time. Cell-specific deletions of astrocytic or neuronal aromatase would be very useful in unraveling these mechanisms. The latter has been used to study synaptic plasticity (75), but to the best of my knowledge, knockout animals lacking aromatase expression in glia remain to be described.

As discussed above, there appears to be ample support for the idea that inflammatory signals can induce aromatase expression. It is unclear if the sex-specific pathways discussed above translate to studies in other species, including mammals. Regardless, in both sexes, there is excellent support for the possibility that E2 can be induced in response to TBI, and as discussed below, that locally synthesized E2 can have dramatic effects on cell turnover, gliosis, and neuroinflammatory condition, among others.

## Astrocytic E2 Provision Is Neuroprotective

The upregulation of aromatase and consequently the increase in neural levels of its product E2 are not trivial. In our hands, a single penetrating injury increases immunoreactive aromatase levels 2- to 3-fold, and local E2 levels 4- to 5-fold, in the injured hemisphere 48 h later relative to the uninjured lobe (67). To the best of my knowledge, this is the most dramatic and rapid change in aromatase expression reported following injury to the vertebrate CNS. Our first attempts at understanding the function of glial aromatization strongly suggested that the upregulation of aromatase in astrocytes following penetrating brain injury was neuroprotective. Site-specific injection of the aromatase inhibitor fadrozole results in greater damage and more gliosis, possible due to increased apoptosis relative the vehicle alone (51, 66). The influence of induced aromatization on indices of degeneration is similar but not identical in the rodent brain. Aromatase expression is induced in astrocytes following various forms of insult in the rodent brain (42, 76–78). In addition, aromatase inhibition following controlled cortical impact in mice results in higher gliosis as measured by the expression of astrocyte-specific markers (18). However, the dramatic injury-induced astrocytic aromatase expression in the finch relative to the murine rodent is perhaps best reflected in the following comparison. In the rodent, despite injury-induced glial E2 provision, the ensuing degeneration demonstrates a clear wave of secondary damage that peaks 24–48 h postinjury (79). In the zebra finch, the inhibitory influence of local aromatization on apoptosis is potent enough to completely mask this wave of secondary degeneration consistently observed in the injured mammalian brain (66). This wave of secondary degeneration, however, is clearly observable upon aromatase inhibition in the injured songbird brain (66). These data suggest that the induction of aromatase is key in controlling brain damage following neural insult in multiple species and highlights the dramatic nature of this response in the songbird.

E2 administration and/or aromatase inhibition with E2 replacement dramatically reverses effects described above with documented decreases in necrosis, gliosis, apoptosis, and injury size in songbirds (49). Further, central E2 provision increases injury-induced cytogenesis and neurogenesis relative to controls (80). In agreement, peripheral or central administration of E2 is neuroprotective in rats and mice [see (81)]. The influence of injury-induced aromatization and E2 provision on multiple indices of cell turnover may reflect the rebuilding of circuits affected by brain damage, including TBI. It is perhaps not surprising that the precise factors that increase glial aromatase expression have been and intense focus of the scientific community in an attempt to develop targeted and specific therapies that ameliorate TBI-associated neural damage and/or accelerate recovery following TBI.

## Injury-Induced Aromatization Is Anti-Inflammatory—Sex-Specific Mechanisms

As mentioned earlier, mechanical damage to the finch brain increases local E_2_ by about 4-fold (67). We hypothesized that elevations in aromatase expression and the consequent rise in local E2 levels may serve as an anti-inflammatory agent via inhibitory actions on the inflammatory cascade. To test this, in individual birds, we compared the levels of various cytokines and enzymes in the inflammatory cascade between hemispheres that were injured in the presence of the aromatase inhibitor fadrozole or vehicle. The results were unequivocal. Across all subjects, 24 h following the injury and drug administration, hemispheres in which the upregulation of aromatase was inhibited with fadrozole showed elevated levels of TNF-α, IL-1β, and COX transcription relative to those that had received vehicle (62). These data support the possibility that local elevations in aromatase activity following injury result in a decrease in several indices of inflammation in male and female zebra finches. This does indeed seem to be the case as the inhibition of injury-induced aromatase via fadrozole also decreased the level of the prostanoid PGE2 relative to the vehicle-treated lobe in both sexes. Taken together, these data point strongly toward local E2 levels as one effector of this anti-inflammatory effect. This possibility was tested in the manner described below.

In a classic replacement experiment, we then tested the levels of cytokine and COX expression in birds where one lobe had been treated with the inhibitor fadrozole (low E2) and the other treated with fadrozole and E2 (replaced E2). Following a 24-h period, hemispheres in which E2 had been replaced had lower levels of certain cytokines (to be discussed later) and COX2 expression relative to the contralateral hemisphere where the expression of aromatase was inhibited without E2 replacement (62). In excellent agreement, E2-replaced hemispheres also had lower levels of PGE2 compared to the fadrozole-treated lobe (62). Thus, injury-induced aromatization serves to control sustained neuroinflammation following penetrating injury in zebra finches and may further protect the brain from the deleterious effects of chronic inflammation. To test the E2 dependency of the effect above, we inflicted bilateral penetrating injuries and injected the aromatase inhibitor fadrozole to adult zebra finches of both sexes. In one hemisphere, however, we concurrently injected E_2_ to assess the potential local influence of this steroid on multiple indices of inflammation (44). We are unaware of similar studies in other animal models and hope to perform similar experiments in nonavian species in the future. We have, however, recently begun probing the mechanism that may underlie the anti-inflammatory effects of E2 in zebra finches.

We followed these studies by examining the mechanism of this action by injuring hemispheres in the presence of ERα or ERβ blockers in both sexes. The results were clear and identical between sexes; whereas E2 continued to demonstrate anti-inflammatory effects in the presence of ERβ antagonist, this effect was completely blocked in the presence of an ERα antagonist (65). These data strongly support an anti-inflammatory role for E2 during brain injury, an effect mediated via ERα receptors in both sexes.

We have long known about the neuroprotective effect of circulating E2 following brain trauma in multiple species. Several studies using *in vivo* preparations and *in vitro* techniques have implicated E2 as an effective protectant across a broad range of neural insults including, but not limited to, excitotoxicity (42, 82), mechanical injury (43, 49, 66), and serum deprivation (83). These findings are in excellent agreement with many data sets supporting a potent anti-inflammatory effect of circulating E2 in multiple species including humans. Indeed, treatment of ovariectomized mice with endotoxin results in larger increases in neural cytokine expression relative to sham controls and ovariectomized mice that have received E2 replacement (84). This pattern is also seen in humans where a decrease in circulating estrogens such as those associated with surgical or natural menopause is coincidental with increases in circulating cytokines [(85); see (86)]. In further support of this anti-inflammatory role, ovariectomized mice demonstrate higher neural cytokine levels upon peripheral endotoxin treatment relative to sham controls [see (78)]. Take together, these data support the notion that estrogens including E_2_ can be anti-inflammatory agents, and this influence extends into neural tissue.

There do appear to be some interesting wrinkles in this story. In our hands, E2 has potent anti-inflammatory effects in both sexes. However, we have documented some interesting sex differences in the influence of E2 on specific components of the inflammatory cascade. While the inhibition of injury-induced aromatase greatly increases several indices of inflammation in females and males (62), including elevations in TNF-α, females appear to also upregulate the expression of IL-1β, whereas males do not. These differences seem to hold true during E2 replacement as well. Specifically, E2 provision during brain injury decreases TNF-α in males, and IL-1β in females. No effect of E2 is observed on male levels of IL-1β or female levels of TNF-α (62). Thus, the initial stages of inflammation appear to be modulated differently by injury-induced aromatization between the sexes. Despite these differences in the initial components of the inflammatory cascade, however, both sexes show dramatic increases in COX expression upon aromatase inhibition, and this is completely ameliorated by replacement with E2 (44, 62). This pattern suggests the possibility that females and males may appropriate different responses to TBI early in the neuroinflammatory cascade, but these differences result in identical downstream signaling further down the biochemical response to inflammation (see Figure 1). We already know that cytokines, while ubiquitous across species, may work differently in females and males (87), and this seems to be true of the neuroinflammatory response to TBI in songbirds. Whether a similar pattern is demonstrated by mammals is currently unknown. However, therapies that seek to harness the anti-inflammatory actions of E2 may prove differentially efficacious between the sexes. It is critical that these differences are documented and understood completely prior to developing potential therapies for all types of TBI.

## Summary and Conclusions

Twenty years of study using the zebra finch as an animal model has provided several important insights into the neuroendocrinology of brain injury. It is noteworthy that the actual incidence of injury-induced aromatase expression following the disruption of the neuropil via a penetrating stab wound is a fairly general phenomenon and has been described in songbirds, rats, and mice by multiple laboratories [see (44)]. It would be interesting to ask if this phenomenon also occurs in humans and other mammalian species. The rapid and dramatic increase of aromatase expression in astrocytes in this species far exceeds that seen in its mammalian counterparts. Not only does local E2 increase at least 4-fold around the site of injury relative to the contralateral hemisphere, but also the upregulation of aromatase responsible for this increase is rapid and/or dramatic enough to completely mask the wave of secondary degeneration observed in the mammalian response to TBI. Interestingly, there seems to considerable feedback between components of the inflammatory response and astrocytic E2 provision in the zebra finch. While the initial response to TBI upregulates prostanoids, which in turn upregulates aromatase and therefore E2, the subsequent action of this E2 provision is a potent downregulation of inflammatory indices. This pattern suggests that the zebra finch may have evolved not only a dramatic response to TBI, but through evolution may have coopted the interactions between inflammation and neurosteroidogenesis to protect vulnerable neural circuits against the deleterious effects of chronic neuroinflammation.

## Author Contributions

The author confirms being the sole contributor of this work and has approved it for publication.

## Conflict of Interest

The author declares that the research was conducted in the absence of any commercial or financial relationships that could be construed as a potential conflict of interest.
